# Evaluating the Impact of the COVID-19 Pandemic on Accessing HIV Services in South Africa: A Systematic Review

**DOI:** 10.3390/ijerph191911899

**Published:** 2022-09-21

**Authors:** Claudia Goncalves Rebelo Jardim, Reza Zamani, Mohammad Akrami

**Affiliations:** 1 Medical School, College of Medicine and Health, University of Exeter, Exeter EX1 2LU, UK; 2 Department of Engineering, University of Exeter, Exeter EX4 4QF, UK

**Keywords:** HIV services, COVID-19 pandemic, South Africa, primary health care, public health, ART, epidemiology, access, systematic review

## Abstract

Progress has been made towards controlling the Human Immunodeficiency Virus (HIV) epidemic in South Africa. However, the emergence of coronavirus disease 2019 (COVID-19) has disrupted access to health care. This systematic review aims to evaluate the impact of the pandemic on accessing HIV services at a primary health care (PHC) level in South Africa. HIV services that have been significantly impacted are highlighted, and recommendations for future public health emergencies are made. Three databases were searched in January 2022. The studies included were those that reported on HIV services at a PHC level in South Africa. From the searches, 203 papers were identified, of which 34 full texts were screened. Eleven studies met the inclusion criteria and were included in this review. Overall, decreases in HIV testing, positive HIV tests, and initiation of antiretroviral therapy (ART) were reported. Resilience of ART provision was reported, meaning that adherence to treatment was sustained throughout the pandemic. The findings showed that HIV services at private PHC facilities were unaffected, however, an overall decrease in HIV services at public PHC facilities was reported, excluding antenatal care which showed resilience.

## 1. Introduction

### 1.1. Background

First identified in the 1980s, human immunodeficiency virus (HIV) has remained highly prevalent, with 37.7 million people worldwide living with the virus. HIV weakens the body’s immune system, leaving individuals vulnerable to opportunistic infections [1]. In 2014, the Joint United Nations Programme on HIV/AIDS (UNAIDS) and its partners, launched the 90-90-90 targets, with the aim of diagnosing 90% of all HIV-positive persons, providing and sustaining antiretroviral therapy (ART) for 90% of those diagnosed, and achieving viral suppression for 90% of those treated, by 2020 [2]. These targets were established to guide efforts to scale up HIV prevention and treatment services [2,3]. Additionally, UNAIDS has also announced the Fast-Track approach, focusing on the 30 countries that account for most new HIV infections, including South Africa [3]. South Africa has an estimated 7.8 million people living with HIV (PLWH), one of the largest HIV epidemics in the world [4].

The history of HIV in South Africa begins during the heightened political tensions of apartheid [5]. In 1985, that the Department of Health launched a controversial HIV/AIDS awareness campaign, together with a number of laws restricting the civil liberties of PLWH. From the 1990s, the country’s legislation began to change, repealing these discriminatory laws and reflecting the transition from apartheid to democracy [5]. In 1993, the first National AIDS plan was published. Although this project was hopeful, years of apartheid privation meant that many Black South Africans lacked access to basic health and social services, and by the mid-1990s, the task of restructuring the economic and political state of the country had taken priority over the fight against HIV [5].

In recent years, South Africa has made progress in combatting HIV. In terms of the 90-90-90 targets, in 2018 it achieved the 90% mark of diagnosing PLWH, and was on track to meet all the targets by 2022. Despite remarkable progress, the prevalence of HIV in the country remains high and is unequally spread [3]. Various factors contribute to HIV prevalence in the country, such as unequal access to health care, poverty, lack of education and high rates of domestic violence [6]. The lack of access to quality health care is a reflection of income inequality in South Africa. This is a result of the socio-political economic system enforced during apartheid, that has now led to disparity in HIV prevalence between genders and among ethnic groups [6]. The HIV epidemic disproportionately affects Black Africans, particularly females, who have the highest prevalence of HIV in the country [6]. This is attributed to the low socio-economic status of women in South Africa, often forcing them to engage in risky sexual behaviour, in addition to high rates of gender-based violence (GBV) [6]. There is also significant spatial variation in the prevalence of HIV, with the highest infection and prevalence rates clustered in the poorest parts of the country [3,7]. With 60.7% of adults living in poverty, KwaZulu-Natal has the highest prevalence of HIV, and the largest number of PLWH in underserved areas [3]. Conversely, the Western Cape has the lowest proportion of adults living in poverty, the lowest number of PLWH and the highest number of healthcare facilities per 1000 PLWH [3,7]. It is clear that socio-economic disparities in health care remain present in post-apartheid South Africa [3,8].

The healthcare system in South Africa has been described as a two-tier system, with a subsidised public sector and a private sector funded by individual payments and health insurance [9]. The public sector provides care to 84% of the population, whilst the private sector provides care to 16% of the population. Both sectors receive similar financial resources, meaning a larger portion of public health sector clients are forced to share this limited resource [10]. Since 1990, the public health system in South Africa has faced a quadruple burden of disease: a dual tuberculosis–HIV epidemic, high maternal and child mortality, and increasing rates of non-communicable diseases [11,12]. Over the past decade, the South African government has increased expenditure on public primary health care (PHC). PHC can be defined as the first point of entry to the healthcare system, provided by general practitioners, nurses and other allied healthcare professionals [13]. PHC facilities reach a large proportion of the population, promoting equitable access to health care. HIV services have been integrated into PHC for this exact reason. The PHC system provides services such as HIV testing, delivery of ART, and ART adherence support. It has been successful in enabling free access to ART for over three million people [14]. There is no effective cure for HIV, however, antiretroviral treatment (ART) is used to manage and reduce the transmission of HIV [1].

### 1.2. Rationale

**Figure 1:**
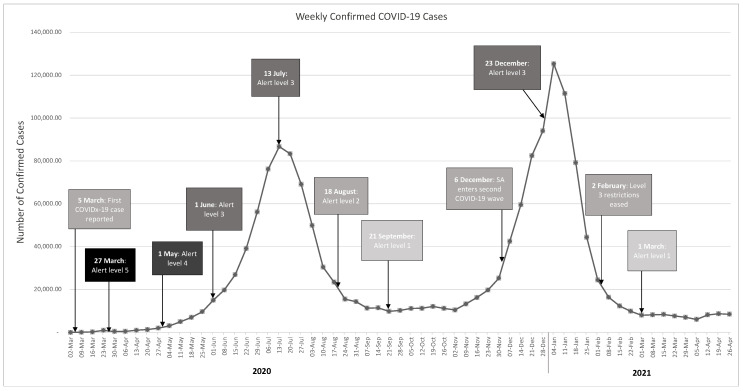
A line graph illustrating the annual number of HIV tests conducted in each province of South Africa, from 2018 to the end of 2020. Data was collected from the National Department of Health website, and represents HIV testing trends in the public health sector [17]. Each point represents the number of HIV tests conducted in the province for that specified year. The trend lines show that between 2018 and 2020, HIV testing in every province increased. In contrast, the trend line for 2020–2021 fell much lower than previous years. No significant change in HIV testing was observed for Free State and the North West, while Gauteng experienced the most drastic decline between 2019–2020 and 2020–2021. This graph has been adapted from Pillay et al. [18].

In 2020, the coronavirus disease 2019 (COVID-19) pandemic emerged, disrupting global HIV-control efforts, and sidelining many routine HIV services to accommodate the response to the pandemic. Figure 1 outlines the timeline of the pandemic in South Africa. In response to the emergence of COVID-19, South Africa entered a strict lockdown from 27 March 2020, that progressed through adjusted levels of severity [15]. The lockdown included restriction of movement, closure of schools and universities, restrictions on social gatherings, and even a ban on the sale of alcohol and cigarettes [15]. A study predicting the potential impact of the pandemic on HIV services suggested that temporary disruptions were likely to affect all aspects of the HIV care cascade, resulting in long-term rises in HIV mortality. It is hypothesised that the lockdown resulted in limited access to and delivery of health care [16]. Despite the emergence of this public health emergency, uninterrupted HIV services at the PHC level remain crucial for reaching goals such as the 90-90-90 target. The effects of the COVID-19 pandemic on HIV services at the PHC level in South Africa remain unclear. This review focuses on highlighting barriers to accessing health care and changes to HIV service delivery amongst different socio-demographic groups during the pandemic.

### 1.3. Objectives

The aim of this study is to evaluate the impact of the COVID-19 pandemic on access to HIV services at a PHC level in South Africa. HIV services include testing, diagnosis, and treatment. Services that were significantly impacted are highlighted. As access to quality health care in South Africa varies according to socio-demographic factors, evidence of changes in service delivery across the public and private health sectors are outlined. We aim to identify where well-informed interventions and policy changes can be made in response to future public health crises, that will not compromise access to routine healthcare services, and will ensure continuity in the PHC system. This study follows the Population–Interest–Context (PICo) format (Table 1).

## 2. Materials and Methods

The methodology of this systematic review follows the PRISMA guidelines [19].

### 2.1. Eligibility Criteria

Table 1 shows the characteristics of studies that were eligible for review. The study design followed the PICo format for a systematic review.

Population: This study investigated patients from all age groups, receiving or seeking to receive HIV services from private or public PHC facilities in South Africa. Studies on PHC workers were also included, as this group is the first point of contact between the community and the healthcare system. HIV services include the processes involved in diagnosing, treating, and preventing HIV, leading to cases being recorded by the national health system. Studies that included no relevant data on HIV services were excluded.

Interest: This was an observational study to evaluate the impact of the COVID-19 pandemic on access to HIV services. Therefore, we searched for cohort studies, article reviews, and meta-analyses with primary data on patient access to HIV services. The impact of COVID-19 covers a range of factors including restricted access due to lockdown measures, redistribution of healthcare resources, and socio-economic challenges due to lockdown.

Context: HIV services were observed at a PHC level, representing the first point of access to health care for the majority of the population of South Africa [13]. The healthcare system in South Africa is a two-tier system, as described above, therefore data on public and private PHC facilities were included, ensuring that patients’ varying socio-economic characteristics were accounted for. PHC facilities include community health centres and clinics. Data from district hospitals were included, as these hospitals form part of a support system for PHC facilities [20]. Studies with data from outside the PHC context were excluded. This review focuses on complete studies published between 2018 and 2022, which limited the search to the most recent data from prior to and during the COVID-19 pandemic period in South Africa. The pandemic period includes the implementation of lockdown, at various levels, from March 2020 to the present day, and was assessed to determine any changes in access to health care during this period. As the study was based in South Africa, papers in a language other than English were excluded.

### 2.2. Search Strategy

The search process was carried out on several dates from January 2022 until the final search on 18 March 2022. Data was collected from the following databases: PubMed, OVID (MEDLINE, Embase, Global Health), and Web of Science. Studies were identified using the following keywords and Medical Subject Heading (MeSH) terms:

1. Health service* OR primary health care access* OR refer*;
2. SARS-CoV-2 OR COVID-19;
3. HIV infection* OR HIV test*;
4. South Africa OR southern Africa.

**Figure A1:**
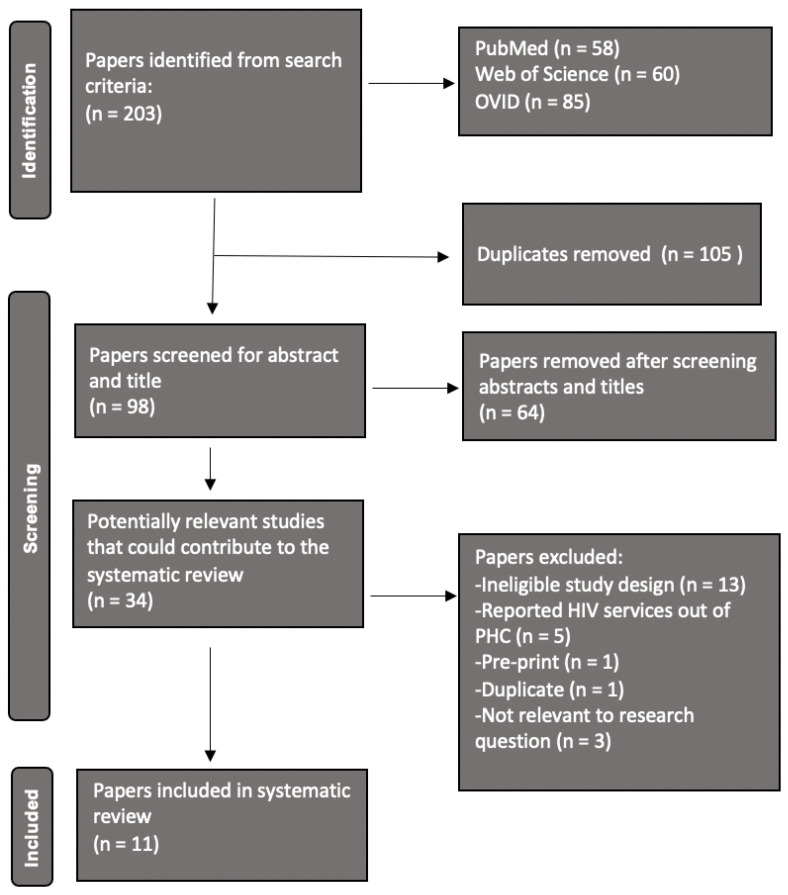
The process of study screening and selection, including the numbers of results from the database searches and numbers and reasons for study exclusion at each stage of the process [19].

Table A1 details the line-by-line advanced searches, including Boolean operators adjusted and applied for each database search. With the Web of Science search terms, to further refine the search, the advanced search field tag “TS” was added to terms in the first three sections of the list above, to ensure that these terms were included in the title and/or abstract of the article. For the OVID and Web of Science search terms, “*” was used to expand the search for changes in suffixes of the word. The searches were limited to publication between January 2018 and January 2022, to obtain the most recent articles and to reflect data on HIV services prior to and during the pandemic. The databases were limited to articles written in English only. After applying the search terms to the databases, a total of 203 studies were suggested for this review. All searches were then exported from the databases to an EndNote20 library, where duplicates were removed. The titles and abstracts of the remaining studies were reviewed, and those not meeting the eligibility criteria were removed. Visual representation of the inclusion and exclusion process in shown Figure A1. A total of eleven studies were suitable for the review as per the eligibility criteria [19].

### 2.3. Study Selection

The first stage of selection included screening the titles and abstracts of articles to determine their relevance to the research question, in accordance with the inclusion and exclusion criteria (Table 1). The studies were then divided into three groups: Include, Exclude and Maybe. Studies in the Include and Maybe groups were then read in full and screened for any relevant data and findings referring to the impact of COVID-19 on HIV services. Authors were contacted for to access any raw data used in their studies. However, no responses were received before this review was completed. Supplementary materials from The Lancet and the South African Medical Journal were included in the review, as they provided data relevant to the research question [21,22].

### 2.4. Quality Appraisal

The quality of the studies included in the review were assessed using the Critical Appraisal Skills Programme (2019) checklist [23]. The CASP checklist was used to critically appraise each study included in the review.

### 2.5. Risk of Bias

A risk of bias may emerge from individual studies included in this systematic review according to the selection of clinic locations and patient data collection methods. This was mitigated by assessing the risk of bias in the individual journals, by considering the journal publication rank using the SCIMago Journal Ranking tool (https://www.scimagojr.com/), accessed on 25 February 2022.

## 3. Results

### 3.1. Search Results

The search of the PubMed, OVID and Web of Science databases produced 203 articles, of which 105 were duplicates. After removing duplicates, the titles and abstracts of 98 articles were screened, in accordance with the eligibility criteria (Table 1). This refined the search to 34 potentially relevant studies that could inform the research. The PRISMA diagram indicates the number of sources eliminated after each review process and highlights the characteristics of those excluded from the review. After reading all articles that were available in full, only eleven studies were included in the final study selection providing data relevant to the research questions. 

### 3.2. Study Characteristics

**Figure 2:**
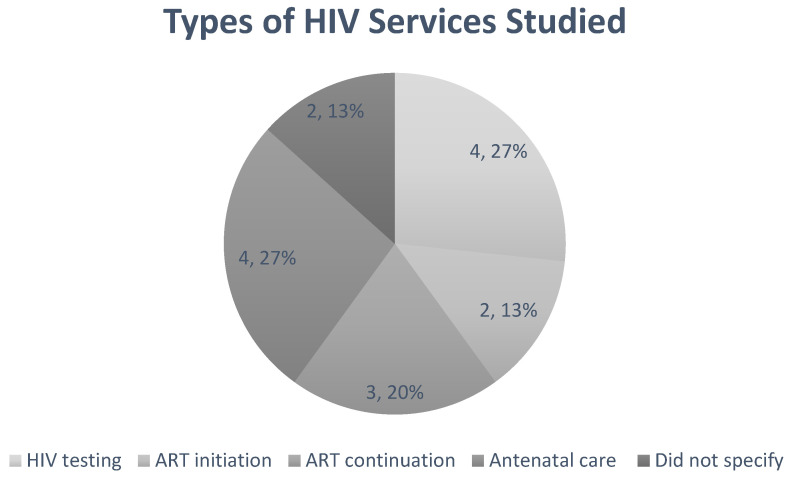
A pie chart representing the distribution of data on HIV services. Approximately 36% of the studies reported on HIV testing, 36% reported on antenatal care, and 27% reported on ART initiation. Only two studies reported on ART initiation. This shows that there is not enough data available for ART initiation, which should be considered as an area of future observation. Two studies did not specify the type of HIV service being reported. (HIV = Human Immunodeficiency Virus; ART = Antiretroviral therapy).

The characteristics and conclusions of the included studies are detailed in Table 2. The sources of the data collected and analysed were considered, as some studies used the same database, explaining their similar findings. The type of HIV service and location of the study were recorded, as these influence the conclusions of the review. Figure 2 highlights the number of papers discussing each type of HIV service. Approximately 36% (4/11) of the studies reported on HIV testing, whilst only two reported on ART initiation. 

### 3.3. Risk of Bias

Most of the studies (54%, 6/11) were published in journals with a ranking of Q2 or Q1. Studies with a ranking of Q3 were included if their findings contributed to the research questions. Although our aim was to include only studies with a ranking of Q2 or higher, some studies with a ranking of Q3 were found to be appropriately designed for the subject matter, and all were peer reviewed.

### 3.4. Result of Individual Studies

#### 3.4.1. Consequences of the COVID-19 Pandemic on HIV Services

All (100%, 11/11) studies reported on the impact of the COVID-19 pandemic on various HIV services. Table 3 and Table 4 outline the chronological changes to HIV services during the pandemic.

Two (18%, 2/11) studies focused on HIV treatment services in the Western Cape during the lockdown period [21,24]. Davey et al. (2020) conducted a cohort study on pre-exposure prophylaxis (PrEP) retention and prescription for pregnant women in Cape Town. During lockdown, 57% of women missed PrEP prescription refill visits, compared to 34% before lockdown [21]. Campbell et al. (2022) used data from questionnaires in the Sinako trial, a cluster randomised controlled trial (RCT) on a household ART adherence intervention for PLWH. At follow-up, 15.66% of respondents stated that they had missed a clinic appointment for HIV care as a result of the lockdown [24]. PLWH who missed an appointment due to lockdown reported lower ART adherence scores. However, there was limited evidence of an association between healthcare access and ART adherence (−1.13, t = −2.69, *p* = 0.009) [24].

Three (27%, 3/11) studies were conducted in clinics in KwaZulu-Natal [25,27,29]. Two of the studies identified resilience in HIV-related clinic visits during the lockdown period [25,29]. The most common reason for clinic visits in 2020 was ART follow-up care, comprising 43% of all visits (*n =* 38,142) [25]. HIV-related clinic visits remained unaffected during the first week of lockdown, and weak evidence of a decrease in the number of ART collection visits was reported for the same time period (IRR 0.932, 95% CI 0.794–1.093) [25,29]. This was followed by a monthly increase in clinic visits, as seen in Table 4 [25,29]. The numbers of missed ART collection visits were noticeably higher in urban clinics (IRR 1.991, 95% CI 1.584–2.503) than in rural clinics (IRR 1.274, 95% CI 1.076–1.509) during the first week of lockdown [29]. Additionally, ART initiations decreased by 46.2% in the first week of lockdown (IRR 0.538, 95% CI 0.459–0.630), and only recovered to 75.3% of pre-lockdown levels (IRR 0.753, 95% CI 0.637–0.890) by mid-June 2020 [29]. The recovery in ART initiations occurred mainly in women (IRR 1.225, 95% CI 1.118–1.341) [29]. With regard to HIV testing, a 47.6% decrease was reported for the first month of lockdown (IRR 0.524, 95% CI 0.446–0.615). However, by July 2020, HIV testing had increased to 82.7% of pre-lockdown levels (IRR 0.827, 95% CI 0.704–0.972) [29]. The median proportion of positive HIV tests per month decreased from 6.1% (IQR 5.4–7.0%) before lockdown to 4.3% (IQR 4.0–4.8%) after lockdown began [29]. Overall, urban clinics reported the largest decreases in clinic visits and HIV testing [29].

Jensen et al., 2021, focused on child health services, reporting significant reductions (*p* = 0.01) in HIV polymerase chain reaction (PCR) testing rates among infants during the first month of lockdown [27]. This is contrasted by findings from two studies reporting resilience in antenatal care, which forms part of the prevention of mother-to-child transmission (PMTCT) programme [18,26]. 

**Figure 3:**
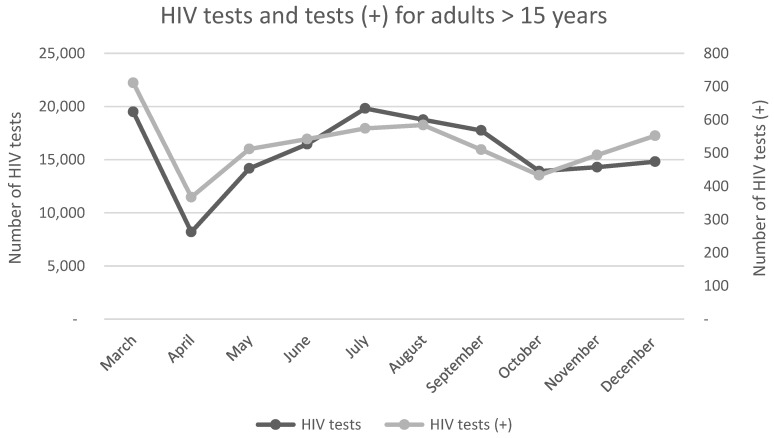
**Line graph presenting the monthly numbers of HIV tests and positive HIV tests (+) for adults over 15 years of age in 2020.** The number of positive HIV tests follows the trend of the number of HIV tests being conducted. A significant decrease in both testing and positive tests was seen for the month of April, coinciding with the start of lockdown level five. A monthly increase in testing and positive tests was seen throughout the year, however, it did not increase to the pre-lockdown level. This figure was created using data from Mutyambizi et al., 2021 [26]. (HIV = Human Immunodeficiency Virus).

**Figure 4:**
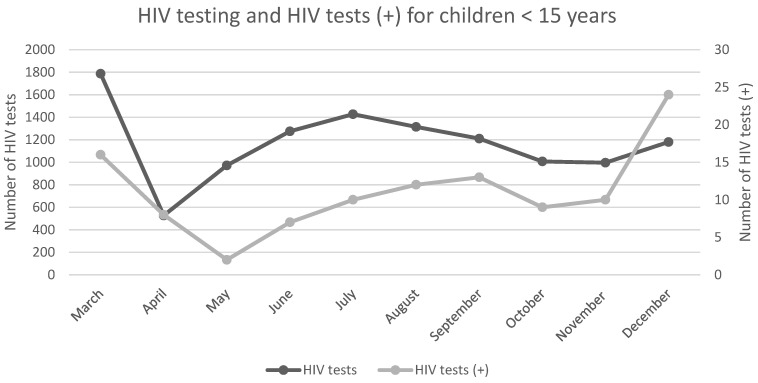
**Line graph presenting the monthly number of HIV tests and positive HIV tests (+) for children under 15 years of age in 2020.** The number of positive HIV tests followed the trend of the number of HIV tests being conducted. A significant decrease in testing and positive tests was seen for the months of April and May, coinciding with the start of the first wave of COVID-19 cases in South Africa. A monthly increase in testing and positive tests was seen throughout the year, however, it did not increase pre-lockdown levels. The number of children < 15 years of age testing positive for HIV significantly increased between November and December. This figure was created using data from Mutyambizi et al., 2021 [26]. (HIV = Human Immunodeficiency Virus).

**Figure 5:**
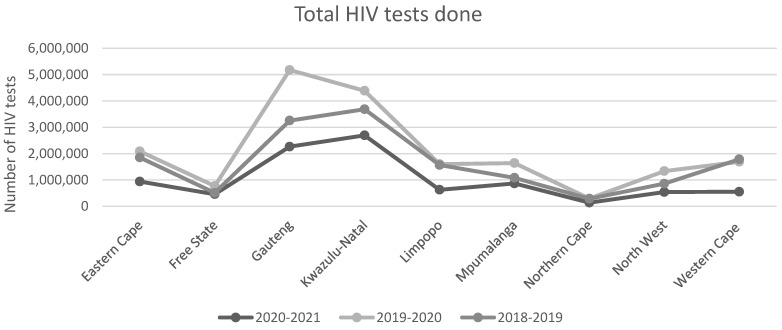
**Line graph illustrating the annual number of HIV tests conducted in each province of South Africa, from 2018 until the end of 2020.** Data was collected from the National Department of Health website, and represents HIV testing trends in the public health sector [17]. Each point represents the number of HIV tests conducted in the province for that specified year. The trend lines show that between 2018 and 2020, HIV testing in every province increased. In contrast, the trend line for 2020–2021 fell much lower than previous years. No significant change in HIV testing can be seen for Free State and the North West, however, Gauteng experienced the most drastic decline between 2019–2020 and 2020–2021.

A study conducted in Limpopo reported a statistically significant (*p* = 0.01) decline in ART initiation for adults and children, over the April–December 2020 period compared with 2019. Children aged <15 years experienced the largest decline in ART initiation (45%) [26]. As seen in Figure 3 and Figure 4, HIV testing decreased for adults (37%) and children (37%) during the first month of lockdown. Significant decreases in positive HIV tests for adults (32%, *p* = 0.01) and children < 15 years (77%, *p* = 0.01) were also reported [26]. As seen in Table 3, this was followed by steady increases in the monthly trend until September 2020. Hereafter, a statistically significant decreases in HIV testing and positive tests were reported for the remainder of the year (*p* = 0.01) [26]. HIV PCR testing remained unaffected during the first month of lockdown, but underwent a significantly decreasing monthly trend thereafter (*p* = 0.022) [26]. Figure 5 illustrates the annual HIV testing volumes within the public health sector, across all nine provinces. National HIV testing within the public health sector declined by 22.3% between March and December 2020, compared with 2019 [18]. The period between April and July 2020, corresponding with lockdown levels five to three, showed the largest decline in HIV testing. The Western Cape reported the largest decline in HIV testing (36.1%) [18]. HIV services in the private health sector remained unaffected by lockdown restrictions and continued at baseline levels throughout most of the pandemic [28].

#### 3.4.2. Patient and Provider Experiences

Table 5 outlines the individual, social, and structural factors that impacted ART adherence during the national lockdown. According to findings, 15.66% of respondents stated that they had missed an appointment for HIV care or treatment as a result of the lockdown [24]. This coincides with the national decline in headcount at public PHC facilities, decreasing from 99.6 million visits in 2019 to 81.2 million in 2020. The Western Cape reported the largest decline in headcount (31.1%) [18]. Most of the studies suggest that the decline in the use of public health services was probably due to the restrictions imposed during the lockdown period, fear of contracting and transmitting COVID-19, and the paucity of healthcare services [18,24,27,29,30]. Despite the decline in attendance at public PHC facilities, Campbell et al. (2021) reported limited evidence of an association between healthcare access and ART adherence (*p* = 0.009) [24].

Moreover, 56.63% of respondents expressed anxiety over running out of HIV medication [24]. In a post hoc analysis conducted by Dorward et al. (2021), there was a higher than usual number of ART collection visits in the four weeks between the first confirmed case of SARS-CoV-2 in South Africa and start of lockdown (IRR 1.233, 95% CI 1.113–1.366), even when seasonality was taken into account [29]. By July 2020, 13.25% of respondents had run out of HIV medication [24]. Participants from the study also reported issues with support from community healthcare workers (CHWs), with 69.88% of participants stating that they had missed support from CHWs [24]. In terms of healthcare provider experiences, Rees et al. (2021) reported the incidence of COVID-19 infections amongst PHC workers providing HIV services. CHWs experienced the highest proportion of confirmed COVID-19 infections amongst PHC workers, with 14% (*n =* 224) of all CHWs having been diagnosed with COVID-19 [30]. More than 5% of staff were on leave due to COVID-19 infection during the five-week period between 26 June–31 July 2020 [30]. Jarolimova et al. (2021) conducted interviews with PHC staff at clinics and community pick-up points. Compared to clinic staff, pick-up-point staff reported significantly lower rates of access to information about performing their work duties during the pandemic (67% vs. 94%; *p* = 0.003), lower perceived preparedness of facilities to work with COVID-19 positive patients (54% vs. 81%; *p* = 0.016), and significantly lower rates of access to necessary protective equipment (50% vs. 89%; *p* < 0.001) [22].

#### 3.4.3. Recommendations and Adaptations of HIV Services

Six studies from multiple authors, including the World Health Organisation (WHO), provided detailed recommendations on how HIV services can be improved or maintained during a pandemic in a high-burden setting. It was suggested that the use of telemedicine could support HIV services during a pandemic. Medical triaging and CHW support could be conducted by telephone [18,21,27,31]. Differentiation of healthcare delivery is recommended, in order to protect vulnerable populations from the risk of COVID-19 infection and to ensure continuity of services. This includes supplying medication through non-medical facilities, and community-based delivery of ART and PrEP [18,21,29,30,31]. The WHO report advocates for at-home HIV self-testing in order to minimise the risk of exposure to COVID-19 [31]. Together with telemedicine, these recommendations would decrease the frequency of visits to healthcare facilities and allow continuity of HIV services during lockdowns [22,25,26]. Emphasis in some studies was also placed on task shifting within the health workforce, and the protection of HIV resources [18,27]. Informing communities of possible service changes and redirecting patients to open facilities are other important recommendations [18]. In order to support ART adherence during a lockdown, the government should facilitate access to food and medication. Access to intimate partner violence (IPV) support services could also be highly beneficial [24].

#### 3.4.4. Implementation of Recommendations 

Four of the included studies documented changes made to HIV services in response to the COVID-19 pandemic. Telemedicine was used for consultations, in the private and public health sector [18,28]. During the first peak of COVID-19 infections, a private healthcare group reported that the monthly percentage of telehealth visits exceeded the monthly average of 10% [28]. Clinics were able to facilitate the provision of ART through multi-month prescribing, and the Central Chronic Medicine Dispensing and Distribution (CCMDD) programme was expanded [18,29]. The CCMDD programme allows PLWH to pick up medication from convenient pick-up points [18]. Reduced operational activity at PHC facilities resulted in patients being sent to district hospitals in order to seek HIV care or treatment. Task shifting at district hospitals was then required as they were expected to provide primary care. Field hospitals were also introduced in order to reduce the emerging pressure on other healthcare facilities [20].

## 4. Discussion

### 4.1. Consequences of the COVID-19 Pandemic on HIV Services

The findings show evidence that the COVID-19 pandemic in South Africa has had a negative impact on certain HIV services. One study, based on a private healthcare group, reported that the lockdown had no effect on HIV services [28]. However, this is not indicative for the entire country as the public sector has a significantly higher burden of HIV due to the social and economic disparities in South Africa. This contrast in findings further emphasises the inequality in access to healthcare, as mentioned in Section 1.1. The majority of the studies reported an overall decrease in HIV services within the public healthcare sector over the lockdown period (27 March–28 December 2020). The studies compared HIV service usage from before the COVID-19 pandemic with that during the lockdown period. Nine provinces in South Africa were reported on, among which were KwaZulu-Natal, Limpopo, and Western Cape. 

In 2020, the main reason for HIV-related clinic visits was for ART follow-up care [25]. Cohort studies based in the Western Cape reported an increase in missed ART follow-up care [21,24]. In contrast, studies based in KwaZulu-Natal identified sustained HIV-related clinic visits during lockdown, specifically for ART collections and follow-up care [25,29]. As previously mentioned, KwaZulu-Natal is one of the most underserved provinces, whilst the Western Cape has a surplus of healthcare facilities per PLWH [3]. Interestingly, the numbers of missed ART collection visits in KwaZulu-Natal were higher in urban than in rural clinics [29]. It can be speculated that either a more stringent lockdown was implemented in urban areas or urban migration had a role to play. The majority of the South African population live in rural areas, and work in urban areas. People who had migrated to urban areas for work might have returned home for lockdown [29]. Ginsburg et al. (2021) found that internal migrants and non-migrants utilise health care differently. Internal migrants are more likely to use private healthcare facilities which are more readily available in urban areas. Additionally, migrants are more likely to be employed and thus able to afford private health care. Migrants utilising private health care at their migration destination were likely to be required to re-engage with rural public health facilities on their return home to rural areas [32]. Further research into HIV services in KwaZulu-Natal during the pandemic would be beneficial for identifying strategies used to ensure the continuity of HIV services.

HIV services that experienced the most significant decreases were ART initiation and HIV testing. According to the Global Fund, countries in Africa and Asia recorded a 41% decline in HIV testing in 2020 versus 2019 [33]. Nationally, HIV testing within the public health sector declined by 22.3% during the 2020 lockdown period, with the largest decline reported in the Western Cape [18]. Three studies reported significant decreases in HIV testing for the first month of lockdown, followed by significant monthly increases [26,27,29]. The overall decrease in testing may have been due to the restriction of movement imposed during the lockdown period, making it difficult for people to attend clinics. Additionally, as mentioned in Section 3.4.2, task shifting amongst CHWs to prioritise COVID-19 patients may have led to fewer referrals for HIV testing [29]. A study in Limpopo reported significant monthly increases in HIV testing up to September 2020. Thereafter, a significant monthly decrease was reported [26]. It can be speculated that the gradual increase in testing occurred due to the easing of lockdown, until September 2020. As seen in Figure 1, a steep rise in COVID-19 cases and the reintroduction of strict lockdown restrictions may have negatively affected HIV testing. HIV PCR testing was unaffected during the first month of lockdown, but significantly decreased in the following months, reflecting the increased demand for COVID-19 PCR testing [26]. This highlights the shift in resources in accordance with demand. A decrease in the number of people testing positive for HIV during the lockdown was reported by two studies [26,29]. The decrease in testing was the same for adults and children. However, the largest decline in positive HIV tests was reported for children < 15 years (77%), which could either be attributed to the maintenance of PMTCT programmes, or the decrease in infant PCR testing [18,26,27,29].

The overall decline in the numbers of positive HIV tests could be linked to the decrease in HIV testing. However, it can also be speculated that social restrictions imposed by the lockdown could have contributed to lower transmission rates of HIV. This hypothesis is challenged by the cohort study in which participants reported no significant change in sexual activity before and during lockdown [21]. Additionally, studies that reported on contraceptive use did not distinguish between types of contraceptives. Further research into social interactions and contraceptive use during the lockdown period would be useful for determining the full impact of the lockdown on HIV transmission.

Decreases in HIV testing and numbers of positive HIV tests led to a significant reduction in the number of people starting ART [18,26,29]. Although Siedner et al., 2021, reported no significant changes in the number of HIV-related clinic visits, which included ART initiation, this is contrasted by two studies reporting a significant decrease in the number of people starting ART [26,29]. An increase in HIV mortality in the coming years has been predicted as a result of this disruption to HIV services [16]. 

### 4.2. Patient and Provider Experiences

The COVID-19 pandemic impacted patients and healthcare providers. As mentioned in Section 3.4.1, sustained ART follow-up care was reported. However, Campbell et al. (2022) concluded that the impact of lockdown on ART adherence was unequal and dependant on a number of individual and social factors [24]. Participants from the study reported feeling anxious about access to health care support their ART adherence, and reported issues with accessing medication [24]. Challenges to healthcare access during lockdown included transport difficulties, fear of contracting and transmitting COVID-19, and paucity of resources [29]. Additionally, a national decline in attendance at public PHC facilities was reported for 2020 [18]. It is hypothesised that people without established patterns of engagement with health care were less likely to overcome the challenges of lockdown to attend clinic visits [29]. A weak association between healthcare access and ART adherence was found. However, that particular study was limited to a small cohort (*n =* 83), thus the association does not reflect national ART adherence [24]. The decrease in ART follow-up care, as mentioned in Section 3.4.1, suggests that national ART adherence was negatively impacted by the pandemic.

Two studies reported that in the four weeks between the first confirmed case of COVID-19 in South Africa and the start of lockdown, there was a higher than usual number of ART collections [25,29]. This infers that PLWH might have prepared for possible stockouts of HIV medication. Participants from the study by Campbell et al. (2022) also reported missing the support offered by CHWs [24]. In some areas, PHC facilities were forced to temporarily close or turn patients away. This was largely due to staff shortages, as high incidence rates of COVID-19 infection amongst CHWs were reported [30]. Additionally, the reallocation of PHC workers to provide services related to COVID-19 limited other healthcare services [18,24,27,29,30]. Currently, CHWs are not well integrated into the healthcare system, with limited experience of personal protective equipment (PPE) and infection prevention and control (IPC) guidelines [22,30]. The pandemic has deterred individuals from health-seeking behaviours and disrupted service delivery, creating a scenario in which treatment regimens are rendered unsuccessful, and providing an opportunity for HIV cases to go undetected, increasing transmission rates in the near future. For future research, better demographic distinctions would be beneficial for highlighting subpopulations who have been more greatly affected. 

### 4.3. Recommendations and Adaptations of HIV Services 

Overall, the studies highlighted the importance of maintaining HIV service provision during a public health emergency. The main recommendation is to prioritise the initiation of ART, by focusing efforts on HIV testing so that individuals can be triaged for treatment. It is suggested that HIV and COVID-19 testing programmes could be integrated in an effort to catch up on testing and treatment [18,29].

Some studies also suggested that the decentralisation of health care could ensure continuity of service delivery [22,25,26]. If differentiated health care is to fulfil its potential, it is also imperative that CHWs are appropriately supported and are well prepared to deliver care safely to patients [22]. Alternative methods of service delivery, such as supplying medication through community-based pick-up points, HIV self-testing, and the use of telephone consultations could be beneficial both to patients and providers [18,21,22,27,29,30]. Furthermore, effective communication of facility closures and resource shortages should be utilised in order for patients to be redirected to available facilities [18]. In terms of social factors, it is proposed that the government should make provisions for adequate access to food, medication, and domestic violence support [24].

### 4.4. Implementation of Recommendations

The findings show evidence of instances where recommendations have been implemented. Telemedicine was utilised in the private and public sectors [18,28]. Further research into the efficiency of telemedicine amongst HIV patients in lower income areas of South Africa would be useful for determining its validity. Chitungo et al. concluded that barriers to the successful implementation of telemedicine in sub-Saharan Africa include poor internet connectivity, insurance companies’ reluctance to pay service providers, and a lack of appropriate mobile devices [34].

Differentiated service delivery was implemented through the CCMDD programme, which was expanded during the pandemic, and the provision of ART was facilitated through multi-month prescription at local clinics [18,29]. This could explain the sustained ART provision that was reported. In Nigeria, a focus was placed on community-based service delivery [35]. Dedicated HIV outreach teams entered communities with a high prevalence of HIV, where they conducted testing, screening, and counselling. The implementation of these strategies may have led to the monthly increase in the number of PLWH in Nigeria initiating ART in 2020 [35].

Patients were redirected to district hospitals as a result of closures at PHC facilities. District hospitals played a crucial role in providing primary care during the pandemic [20]. In order to adapt to the pressure on the healthcare system, field hospitals were built to support surrounding facilities. Patients requiring ongoing care were transferred to these facilities, creating more availability at district hospitals [20]. Further research into the effectiveness of these additional facilities is required to assess their impact in this high-burden setting.

### 4.5. Strengths and Limitations

The main strength of this review is that it is the first to date to outline evidence of disruptions to HIV services at a PHC level during the COVID-19 pandemic in South Africa. It also reports changes in HIV services in two provinces with contrasting economic and healthcare characteristics, comparing service disruptions between different socio-economic groups. 

The major limitation of this review is that 36% (4/11) of the studies reported on data from a single source—the District Health Information System (DHIS). Routine DHIS data collection is subject to human error, and the inclusion of other data sources would highlight any discrepancies. Another limitation is that this review was only able to report on three out of the nine provinces of South Africa. Data from the other provinces would be beneficial for conclusions to be made for the country as a whole. 

Furthermore, considering the evolving nature of the pandemic, it is likely that the emergence of new variants and the prioritisation of the COVID-19 vaccination programme might also impact HIV services. Future research into the impact of the vaccination programme on HIV services would be useful. 

## 5. Conclusions

This review has found an association between the COVID-19 pandemic and a decrease in certain HIV services at a PHC level. No changes to HIV services at private PHC facilities were found. However, significant decreases in HIV testing, positive HIV tests, and ART initiation at public PHC facilities were reported. This confirms the disparity in PHC access between the private and public sectors. The decrease in HIV testing can be attributed to several factors, including lockdown restrictions, staff shortages at facilities, and scarcity of resources. A barrier to accessible health care was created, which led to a decrease in HIV testing. As fewer people were tested, this concurrently decreased the number of people testing positive for HIV. It might also be inferred that social restrictions during lockdown lowered the transmission of HIV, making this an important area for further research. It is interesting to mention that children saw the greatest decline in numbers of positive HIV tests, highlighting the resilience of the PMTCT programmes during the pandemic [18,26]. The decrease in people starting ART can confidently be linked to the decreases in HIV testing and positive HIV tests [18,26,29]. It is important that individuals are tested and diagnosed in order to begin ART. Recommendations prioritising HIV testing were made by studies included in the review [18,29]. Resilience in ART adherence differed between KwaZulu-Natal and the Western Cape. KwaZulu-Natal showed resilience in ART follow-up care, whilst the Western Cape saw a decrease. This is significant as these provinces differ in terms of healthcare capacity and socio-demographic characteristics [3,7]. Further research would be beneficial to better understand the strategies utilised in KwaZulu-Natal to ensure continuity of services. Overall, the reported decline in testing and initiation of treatment is set to hinder South Africa’s goal of reaching the UNAIDS 90-90-90 targets by 2022, and will create a backlog of undiagnosed PLWH. The PHC system in South Africa must be strengthened, and should address the issues caused by COVID-19 in order to create effective strategies for future pandemics.

## Figures and Tables

**Table 1:** Inclusion and exclusion criteria.

	Inclusion Criteria	Exclusion Criteria
Population	Patients from all age groups receiving HIV services from: Private and public primary healthcare facilities in SA Primary healthcare providers	HIV services outside the primary healthcare system
Interest	Inequalities in access to HIV care services during the COVID-19 era	Biomarker detection in the blood
Context	Impact on HIV care services Studies conducted in SA	Studies without SA-based data
Study design	Cohort studies	Non-English studies Studies published before 2017

Our study follows the Population–Interest–Context (PICo) format.

**Table 2:** Study Characteristics.

Author and Date	Aim of Study	Location	Journal Rank	Data Source	Type of HIV Service(s) Reported	Study Conclusion
[22]	To investigate changes in individual, social, and structural factors during the COVID-19 pandemic, and whether these changes impacted ART adherence during the lockdown.	Cape Metro area, Western Cape	Q1	Base-line and follow-up data from participants in the Sinako trial (31)	- Adherence to ART	- The impact of lockdown is unequal, and is dependent on a combination of individual, social and structural factors. - Association between positive household environment and good ART adherence.
[19]	To evaluate the effect of the COVID-19 pandemic on PrEP-PP study visits and on PrEP prescription refill visits among pregnant women in antenatal care	Cape Town, Western Cape	Q1	Cohort study	- PrEP prescription refill visits	- During lockdown, missed PrEP visits increased significantly by 63% at the 1-month visit, and 55% at the 3-month visit. - Overall, 57% of women missed their PrEP visits during lockdown.
[23]	To measure the impact of the COVID-19 lockdown on HIV testing and treatment	Kwazulu-Natal	Q1	District Health Information System (DHIS)	- Testing for HIV - Initiating ART - ART collection	- In rural areas, HIV services were generally maintained for people already receiving ART. - Engaging new people into care was impeded by the lockdown, particularly in urban clinics.
[24]	To assess the impact of the COVID-19 outbreak on routine child health services	Kwazulu-Natal	Q3	District Health Information System (DHIS)	- HIV PCR testing at birth	- Significant reductions in infant PCR testing for HIV, large variations in data indicate inequalities in service delivery.
[18]	To evaluate the characteristics, clinical management and outcomes of patients with COVID-19 at district hospitals	Western Cape	Q1	Medical records from eight district hospitals	-	- District hospitals provided essential primary care service during lockdown, whilst access to primary care facilities was limited.
[25]	To analyse trends in HIV, TB and PMTCT indicators during the COVID-19 lockdown	Mopani district, Limpopo	Q3	District Health Information System (DHIS)	- HIV tests - HIV tests (+) - ART initiation - ART adherence - Antenatal visits	- HC, HIV, and ART indicators were negatively affected by lockdown. - PMTCT and TB indicators were mostly unaffected.
[26]	To assess the direct and indirect effects of the COVID-19 pandemic on private healthcare utilisation	All nine provinces	Q1	Data provided by *Netcare* and *Medicross*	- GP visits for HIV	- GP consultations for HIV were not substantially affected by lockdown restrictions, and continued at baseline levels throughout most of the pandemic.
[27]	To assess the impact of COVID-19 on routine primary healthcare services	All nine provinces	Q3	District Health Information System (DHIS)	- Access to contraceptives and family planning - HIV testing	- All provinces experienced a decline in PHC headcount, WC had the largest decline of 31.1%. - The largest decline in the prescription of contraceptives was seen between April and May 2020 (highest level of lockdown). - A 22.3% decline in national HIV testing between March and December 2020.
[28]	To analyse the rates of COVID-19 infection amongst Anova-employed HCWs	Cape Town, Western Cape Johannesburg, Gauteng Capricorn and Mopani, Limpopo Sedibeng, Gauteng	Q3	Employee database	- HIV testing amongst PHC workers	- CHWs are at higher risk of COVID-19 infection due to insufficient training and poor infrastructure. - Staff shortages were largely due to the high infection rates amongst CHWs.
[29]	To evaluate whether the implementation of lockdown affected access to PHC	Northern Kwazulu-Natal	Q1	Agincourt health and socio-demographic surveillance system (HDSS)	- Initiation of ART - Continuation of ART - ART collection under the chronic care medical dispending programme	- Evidence of sustained visitation in HIV ambulatory clinic utilisation. - Estimated 20% increase in clinic visits for HIV immediately after lockdown.
[20]	To evaluate the effectiveness of decentralised HIV care during the COVID-19 pandemic	Kwazulu-Natal	Q3	Interviews with 112 clinic staff and 24 pick-up point staff	- Preparedness amongst PHC workers at clinics and community-based pick-up points to deliver ART	- Pick-up point staff and facilities were inadequately prepared to work with HIV patients during the COVID-19 pandemic.

This summary reports on the aim, location, and journal rank of the studies. Each study’s data source is included, as this accounts for similarities in some of the findings. Journals are ranked into quartiles: Q1 being of the highest quality and Q4 being the lowest. This was conducted following the inclusion and exclusion criteria to assess the quality of the papers. Those with a Q4 ranking were excluded and those in the Q3 group were assessed based on the value of the information in the paper. ‘-’ = missing data; COVID-19 = Coronavirus disease 2019; ART = Antiretroviral Therapy; PrEP = Pre-exposure Prophylaxis; HIV = Human Immunodeficiency Virus; PCR = Polymerase Chain Reaction; TB = Tuberculosis; PMTCT = Prevention of Mother-to-Child Transmission; HIV test (+) = Positive HIV test; HC = Headcount; WC = Western Cape; CHW = Community Healthcare Worker; HCW = Healthcare Worker; PHC = Primary Health Care; CCMDD = Central Chronic Medicine Dispensing and Distribution Programme.

**Table 3:** This table summarises the changes in HIV testing services in chronological order, from before lockdown, to during lockdown. Data are expressed by incidence rate ratio (CI 95%), or the interquartile range is given. (IQR = Interquartile Range; IRR = Incidence Rate Ratio; CI = Confidence Interval; HIV = Human Immunodeficiency Virus; PCR = Polymerase Chain Reaction).

Reference	1–26 March 2020	26 March–30 April 2020	1–31 May 2020	1 June–17 August 2020	18 August–21 September 2020	21 September–28 December 2020
	Pre-Lockdown	Level 5	Level 4	Level 3	Level 2	Level 1
[22]	HIV tests positive per month are 6.1% (IQR 5.4–7.0%)	47.6% decrease in HIV testing (IRR 0.524, 95% CI 0.446–0.615)		HIV testing reaches 82.7% of pre-lockdown levels (IRR 0.827, 95% CI 0.704–0.972)		
		HIV tests positive per month are 4.3% (IQR 4.0–4.8%)				
[23]		Infant PCR testing at birth declined by 5% in March, and 6% in April (*p* = 0.01)	Infant PCR testing at birth declined by 15% (*p* = 0.01)	Infant PCR testing at birth declined by 15% (*p* = 0.01)		
[27]		Significant decrease in HIV testing for the age group 18 months–14 years (*n* = 665; *p* = 0.05)	Statistically significant increase in the monthly trend for HIV testing (*p* = 0.031) and HIV tests (+) (*p* = 0.003) for age groups 18 months–14 years			Significant decrease in HIV testing for the age group 18 months–14 years (*n* = 697; *p* = 0.01)
		Significant decrease in HIV testing for age group ≥ 15 years (*n* = 10,592; *p* = 0.01)	Highly significant increase in HIV testing and positive HIV tests for age group ≥ 15 years (*p* < 0.001)			Significant decrease in HIV testing for age group ≥ 15 years (*n* = 7056; *p* = 0.01)
		PCR testing and PCR test (+) are unaffected				
		Significant decrease in HIV test (+) for the age group 18 months–14 years (*n* = 22; *p* = 0.01)	Statistically significant decreases in PCR testing (*p* = 0.022) and no significant change to PCR tests (+) (*p* = 0.258)			Significant decrease in HIV test (+) for the age group ≥ 15 years (*n* = 195; *p* = 0.01)
		Significant decrease in HIV test (+) for the age group ≥ 15 years (*n* = 277; *p* = 0.01)				
[29]		Largest national decline in HIV testing				

**Table 4:** This table summarises the changes in ART services in chronological order, from before the lockdown, to during the lockdown. Some data are in incidence rate ratio (CI 95%) or the interquartile range is given. (IQR = Interquartile Range; IRR = Incidence Rate Ratio; CI = Confidence Interval; HIV = Human Immunodeficiency Virus; ART = Antiretroviral Therapy).

Reference	1–26 March 2020	26 March–30 April 2020	1–31 May 2020	1 June–17 August 2020	18 August–21 September 2020	21 September–28 December 2020
	Pre-Lockdown Period	Level 5	Level 4	Level 3	Level 2	Level 1
[22]	Increase in number of ART collection visits (IRR 1.233, 95% CI 1.113–1.366)	46.2% decrease in ART initiations (IRR 0.538, 95% CI 0.459–0.630)		ART initiations reach 75.3% of pre-lockdown levels (IRR 0.753, 95% CI 0.637–0.890)		
		Weak evidence of a small decrease in number of ART collection visits (IRR 0.932, 95% CI 0.794–1.093)		Some evidence suggests ART collection visits were lower than pre-lockdown levels (IRR 0.859, 95% CI 0.747–0.989)		
[27]		45% decrease in ART initiation for the age group < 15 years	Statistically significant decreases in adults and children initiating ART			No recovery in ART initiation rate
		33% decrease in ART initiation for treatment-naïve adults				
		41% decrease in ART initiation for treatment-naïve children naïve				
		1% decrease in ART continuation for children < 15 years				
[24]	37.6 mean daily HIV-related clinical visits	45.5 mean daily HIV-related clinical visits, an estimated 20% increase	56.6 mean daily HIV-related clinical visits	60.6 mean daily HIV-related clinical visits		

**Table 5:** Social factors impacting ART adherence.

Social, Structural or Individual Factor	Impact of COVID-19 on Social, Structural or Individual Factors	Impact on ART Adherence
Healthcare access	A minority (15.66%) of participants reported issues with accessing health care to support their ART adherence.	There was limited evidence of an association between healthcare access and ART adherence (−1.30, t = −2.69, *p* = 0.009).
Food insecurity	The majority of participants (60%) reported issues obtaining food during lockdown.	Participants who stated that in their household, adult food consumption was often restricted reported lower ART adherence scores (−1.19, Z = −2.33, *p* = 0.02).
Economic insecurity	78.95% of participants reported falling below the poverty line.	There was no evidence of an association between ART adherence scores and household income (0.13, t = 0.29, *p* = 0.78).
Household HIV stigma	A decrease in household HIV stigma was reported, with 50% of participants reporting that they do not feel blamed by their household members because of their HIV status.	Experiencing stigma was negatively associated with ART adherence scores (−4.06, t = −2.86, *p* = 0.002).
Household violence	A decrease in household violence was reported, with 92.77% of participants reporting no instances of household violence following lockdown.	There was strong evidence that experiencing all forms of violence was associated with lower ART adherence scores (−2.09, t = −2.55, *p* = 0.01).
Household functioning	Following lockdown, more participants (95.18%) reported a feeling of togetherness in their household.	Participants whose household members did not work together to work out problems reported lower ART adherence scores (−3.02, t = −3.01, *p* = 0.004).
Self-reported wellbeing	Over a quarter of participants reported feeling more depressed since the arrival of COVID-19 (29%), and since the start of lockdown (27%).	Participants feeling depressed during the lockdown reported lower ART adherence scores (−1.17, t = −2.47, *p* = 0.02).

## Data Availability

Not applicable.

## Abbreviations

**ART**	Antiretroviral Therapy
**AIDS**	Acquired Immune Deficiency Syndrome
**CASP**	Critical Appraisal Skills Programme
**CCMDD**	Central Chronic Medicine Dispensing and Distribution
**CHW**	Community Healthcare Worker
**COVID-19**	Coronavirus disease 2019
**DHIS**	Department of Health Information System
**GBV**	Gender-based Violence
**HCW**	Healthcare Worker
**HIV**	Human Immunodeficiency Virus
**IPC**	Infection Prevention and Control
**PCR**	Polymerase Chain Reaction
**PHC**	Primary Health Care
**PiCo**	Population, Interest, Context
**PLWH**	People Living With HIV
**PMTCT**	Prevention of Mother-to-Child Transmission
**PPE**	Personal Protective Equipment
**PrEP**	Pre-exposure Prophylaxis
**UNAIDS**	United Nations Programme on HIV/AIDS
**WHO**	World Health Organisation

## Appendix A

**Table A1:** Line-by-line search history.

	PubMed	Web of Science	Ovid (MEDLINE)
**Search terms**	(“Health Services”[Mesh] OR “primary health care”[Mesh] OR “health care”[tiab] OR “health service*”[tiab] OR refer*[tiab] or access*[tiab])	TS = (“Health service*” OR “primary health care” OR “primary AND healthcare” OR “primary AND care” OR refer* OR access*)	(Health services OR primary health care OR primary healthcare OR health service* OR access* OR refer*).ti,ab.
	AND	AND	AND
	(“SARS-CoV-2”[Mesh] OR “COVID-19”[Mesh] OR covid[tiab] OR coronavirus[tiab] OR “corona virus”[tiab])	TS = (SARS-CoV-2 OR COVID-19 OR covid OR coronavirus OR “corona AND virus”)	(COVID-19 OR SARS-CoV-2 OR coronavirus).ti,ab.
	AND	AND	AND
	(“HIV Infections”[Mesh] OR HIV[tiab] OR “HIV test*”[tiab] OR “HIV diagnos*”[tiab])	TS = (“human immunodeficiency virus” OR HIV OR “HIV infection*” OR “HIV test*” OR “HIV diagnos*”)	(HIV OR HIV infection* OR HIV test* OR HIV diagnos*).ti,ab.
	AND	AND	AND
	(“South Africa”[tiab] OR “southern Africa”[tiab])	CU = (“South Africa” OR “southern Africa”)	(South Africa OR southern Africa).ti,ab.
**Filters applied**	From 2018–2022	From 2018–2022	From 2018–2022
	English	English	English

tiab = title or abstract; Mesh = Medical Subject Heading; “*” indication truncation, searching for variation of suffixes and prefixes. TS = topic; CU = country/region. ti,ab. = title or abstract. The Boolean operators ‘AND’ and ‘OR’ were applied to combine searches and further refine the database search. The same terms were used for each database to ensure consistency in the search results. All searches were limited to publication dates between 2018 and 2022. English language was selected for the search.
